# The relation between smokeless tobacco and cancer in Northern Europe and North America. A commentary on differences between the conclusions reached by two recent reviews

**DOI:** 10.1186/1471-2407-9-256

**Published:** 2009-07-29

**Authors:** Peter N Lee, Jan Hamling

**Affiliations:** 1 P.N. Lee Statistics and Computing Ltd, Surrey, UK

## Abstract

**Background:**

Smokeless tobacco is an alternative for smokers who want to quit but require nicotine. Reliable evidence on its effects is needed. Boffetta et al. and ourselves recently reviewed the evidence on cancer, based on Scandinavian and US studies. Boffetta et al. claimed a significant 60–80% increase for oropharyngeal, oesophageal and pancreatic cancer, and a non-significant 20% increase for lung cancer, data for other cancers being "too sparse". We found increases less than 15% for oesophageal, pancreatic and lung cancer, and a significant 36% increase for oropharyngeal cancer, which disappeared in recent studies. We found no association with stomach, bladder and all cancers combined, using data as extensive as that for oesophageal, pancreatic and lung cancer. We explain these differences.

**Methods:**

For those cancers Boffetta et al. considered, we compared the methods, studies and risk estimates used in the two reviews.

**Results:**

One major reason for the difference is our more consistent approach in choosing between study-specific never smoker and combined smoker/non-smoker estimates. Another is our use of derived as well as published estimates. We included more studies, and avoided estimates for data subsets. Boffetta et al. also included some clearly biased or not smoking-adjusted estimates. For pancreatic cancer, their review included significantly increased never smoker estimates in one study and combined smoker/non-smoker estimates in another, omitting a combined estimate in the first study and a never smoker estimate in the second showing no increase. For oesophageal cancer, never smoker results from one study showing a marked increase for squamous cell carcinoma were included, but corresponding results for adenocarcinoma and combined smoker/non-smoker results for both cell types showing no increase were excluded. For oropharyngeal cancer, Boffetta et al. included a markedly elevated estimate that was not smoking-adjusted, and overlooked the lack of association in recent studies.

**Conclusion:**

When conducting meta-analyses, all relevant data should be used, with clear rules governing the choice between alternative estimates. A systematic meta-analysis using pre-defined procedures and all relevant data gives a lower estimate of cancer risk from smokeless tobacco (probably 1–2% of that from smoking) than does the previous review by Boffetta et al.

## Background

In 2008, Boffetta et al. [1] published a short review in Lancet Oncology of the evidence relating smokeless tobacco (ST) to cancer. Included was a table summarizing smoking-adjusted relative risk (RR) estimates with 95% confidence intervals (CI) relating to cancer of the oral cavity, oesophagus, pancreas and lung in the USA and Northern Europe taken from 18 studies, together with a further table of meta-analysis results. The results of the overall (USA and Nordic countries combined) meta-analyses are summarized in Table 1, and show a statistically significant increase of 60–80% for ever smokeless tobacco use for oral, oesophageal and pancreatic cancer, and a non-significant 20% increase for lung cancer. Results for other cancers were stated to be "too sparse for a quantitative investigation."

**Table 1:** Comparison of our smoking-adjusted random-effects meta-analysis estimates with those of Boffetta et al.

	Boffetta et al. [1]		Lee and Hamling [2]	
		
Cancer	N^a^	RR (95% CI)^b^	N^a^	RR (95% CI)^b^
Oropharyngeal	13	1.8 (1.1–2.9)	19	1.36 (1.04–1.77)
- published since 1990		Not given	14	1.00 (0.83–1.20)
Oesophageal	5	1.6 (1.1–2.3)	7	1.13 (0.95–1.36)
Pancreatic	6	1.6 (1.1–2.2)	7	1.07 (0.71–1.60)
Lung	5	1.2 (0.7–1.9)	6	0.99 (0.71–1.37)
Stomach		Not given	8	1.03 (0.88–1.20)
Bladder		Not given	10	0.95 (0.71–1.29)
Overall cancer		Not given	7	0.98 (0.84–1.15)

^a ^Number of individual estimates considered in meta-analysis.

^b ^Smoking-adjusted estimates for any ST use.

In their review Boffetta et al. [1] give only limited information on their "search strategy and selection criteria." While they make it clear that they restricted attention to papers published up to September 2007 (including one in press at that time) they give little information on how they selected the cancers for detailed study or how they chose the estimates to be included in their meta-analyses. Thus they note that results for cancers other than those of the oral cavity, oesophagus, pancreas, and lung were "too sparse for quantitative information" without specifying the amount of data needed for analysis. Furthermore they state merely that "we included only studies restricted to non-smokers and studies that included smokers but were properly adjusted for the possible confounding effect of tobacco smoking." without giving any indication as to how they chose from alternative estimates available in a number of the papers (e.g. by sub-type of cancer, type of smoking adjustment, type of ST or timing of ST exposure). A meticulous description of the methods used should have been included, but was not.

Shortly before the review of Boffetta et al. [1] was published, we had started our own review of this evidence, a review which has recently been published in BMC Medicine [2]. We continued with our review, because our initial impression of Boffetta et al.'s was that some relevant data had been missed and that some of the RRs used in their meta-analyses seemed inappropriate. Although our review also considered effect estimates that were not adjusted for smoking, we took particular care to distinguish those that were adjusted for smoking. Our smoking-adjusted meta-analysis estimates are also shown in Table 1. As will be seen, our estimates are substantially lower for all four cancers considered by Boffetta et al. For oesophageal, pancreatic and lung cancer the estimated increases are all less than 15% and not statistically significant, while for oral cancer our estimate of a 36% increase, though statistically significant, is lower than the 80% increase estimated by Boffetta et al., and disappears when attention is restricted to studies published since 1990. Our review also considers a range of other cancers, and Table 1 also presents meta-analysis estimates for stomach, bladder and overall cancer. Each is based on at least as many RRs as are available for oesophageal, pancreatic and lung cancer, and none shows a significant excess risk in ST users.

### Objectives

The objective of this article is to provide a detailed comparison of the two reviews [1,2] in order to clarify why these major differences in risk estimates have occurred. Attention is restricted to the four cancers considered by Boffetta et al. [1]

### Differences between the estimates from the two reviews

Table 2 (oropharyngeal cancer [3-21]), Table 3 (oesophageal cancer [4,18,22-25]), Table 4 (pancreatic cancer [4,5,26-30]) and Table 5 (lung cancer [3-5,27,31]) summarize the estimates used in the two reviews [1,2], with comments on similarities and differences. Based on this comparison, the details of the methodology given in our review [2], and the rather brief description of their procedures presented by Boffetta et al. [1], a number of general observations can be made.

**Table 2:** Comparison of individual and overall (random-effects) estimates for the two reviews – oropharyngeal cancer

	ST use^a^						
						
Ref	Type	Exposure	Inclusion of smokers^b^	Review^c^	Sex	Relative risk (95% CI)	Comments
[3]	ST	Current	NS	L&H	M	2.02 (0.53–7.74)	
(CPS-I)	ST	Current	NS	B	M	2.0 (0.5–7.7)	Estimates agree^d^
							
[3]	ST	Current	NS	L&H	M	0.90 (0.12–6.71)	
(CPS-II)	ST	Current	NS	B	M	0.9 (0.1–6.7)	Estimates agree^d^
							
[4]	Snuff	Ever	SNS	L&H	M	1.10 (0.50–2.41)	
	Snuff	Ever	SNS	B	M	1.1 (0.5–2.4)	Estimates agree^d^
							
[5]	Snuff	Ever	SNS	L&H	M	0.7 (0.5–0.9)	
	Snuff	Ever	NS	B	M	0.8 (0.4–1.7)	NS not SNS
							
[6]	Snuff	Ever	SNS	L&H	M	3.1 (1.5–6.6)	Too recent to be included by B
							
[7]	Chew	Ever	SNS	L&H	M+F	2.05 (1.48–2.83)^e^	Not included by B
							
[8]	Chew	Ever	SNS	L&H	M	2.00 (1.16–3.47)^e^	Not included by B
							
[9]	ST	Ever	SNS	L&H	M	3.63 (1.02–12.95)^e^	Not included by B
							
[10]	Snuff	Ever	SNS	L&H	F	2.67 (1.83–3.90)^e^	
	Snuff	Ever	NS^f^	B	F	4.2 (2.6–6.7)	Whites
	Snuff	Ever	NS^f^	B	F	1.5 (0.5–4.8)	Blacks
							
[11]	ST	Ever	SNS	B	M	2.3 (0.2–12.9)	Tongue cancer
	ST	Ever	SNS	B	M	11.2 (4.1–30.7)	Mouth cancer
							Not included by L&H as no valid smoking adjustment^g^
							
[12]	ST	Ever	NS	L&H	F	6.2 (1.9–19.8)	
	ST	Ever	NS	B	F	6.2 (1.9–19.8)	Estimates agree
							
[13]	Snuff	Ever	NS	L&H	M+F	0.67 (0.08–5.75)^e^	Not included by B
							
[14]	ST	Ever	SNS	L&H	M+F	1.04 (0.41–2.68)^e^	Not included by B
							
[15]	ST	Ever	SNS	L&H	M	0.96 (0.70–1.33)^e^	
	Chew	Ever	SNS	B	M	1.0 (0.7–1.4)	Chew not ST
							
[16]	Chew	Ever	SNS	L&H	M	1.11 (0.81–1.53)^e^	
	Chew	Ever	NS	B	M	2.3 (0.7–7.3)	NS not SNS
							
[17]^h^	ST	Ever	SNS	L&H	M+F	1.43 (0.64–3.21)^e^	Not included by B
							
[18]	Snuff	Ever	SNS	L&H	M	0.98 (0.63–1.50)^e^	
	Snuff	Ever	SNS	B	M	1.4 (0.8–2.4)	Oral cancer excluding pharynx
							
[19]	Snuff	Ever	SNS	L&H	M+F	0.8 (0.5–1.3)	Estimate for NS also available
	Snuff	Ever	SNS	B	M+F	0.8 (0.5–1.3)	Estimates agree
							
[20]	ST	Ever	SNS	L&H	M	1.0 (0.4–2.3)	Not included by B
							
[21]	Snuff	Ever	SNS	L&H	M+F	0.7 (0.3–1.3)	Not included by B
							
Total				L&H		1.36 (1.04–1.77)	19 estimates
				B		1.8 (1.1–2.9)	13 estimates

^a ^ST = smokeless tobacco; Chew = chewing tobacco; ever exposure includes undefined use.

^b ^NS = never smokers; SNS = smokers and nonsmokers combined (with adjustment for smoking).

^c ^L&H = Lee and Hamling review [2]; B = Boffetta et al. review [1].

^d ^To within rounding error, as B only expressed estimates to one decimal place.

^e ^Estimated from data provided.

^f ^B stated that the results were for never smokers, but L&H consider the result relates to non-current smoking. L&H's estimate is for current and non-current smokers combined.

^g ^Valid smoking adjustment was impossible in this study as "for users of multiple tobacco products, only the primary product was recorded".

^h ^The results were cited by Gross et al. [17] based on an unpublished report by Perry et al., "Attributable oral cancer risk due to smokeless tobacco use based on a case-control study at Sinai Hospital in Detroit".

**Table 3:** Comparison of individual and overall (random-effects) estimates for the two reviews – oesophageal cancer

	ST use^a^						
						
Ref	Type	Exposure	Inclusion of smokers^b^	Review^c^	Sex	Relative risk (95% CI)	Comments
[4]	Snuff	Ever	SNS	L&H	M	1.40 (0.61–3.24)	
	Snuff	Ever	SNS	B	M	1.4 (0.6–3.2)	Estimates agree^d^
							
[22]	Snuff	Ever	SNS	L&H	M	1.00 (0.79–1.27)^e^	Estimate for NS also available
	Snuff	Ever	NS	B	M	3.5 (1.6–7.6)	NS not SNS; squamous cell carcinoma not all oesophageal cancer
							
[23]	Chew	Ever	NS	L&H	M	1.18 (0.28–4.90)^e^	Not included by B
	Chew	Ever	NS	L&H	F	2.69 (0.92–7.87)^e^	Not included by B
							
[24]	ST	Ever	NS	L&H	M	1.2 (0.1–13.3)	
	ST	Ever	NS	B	M	1.2 (0.1–13.3)	Estimates agree
							
[18]	Snuff	Ever	SNS	L&H	M	1.2 (0.7–2.2)	
	Snuff	Ever	SNS	B	M	1.2 (0.7–2.2)	Estimates agree
							
[25]	Snuff	Ever	SNS	L&H	M+F	1.31 (0.89–1.92)^e^	
	Snuff	Ever	SNS	B	M+F	1.4 (0.9–2.3)	Squamous cell carcinoma not all oesophageal cancer
							
Total				L&H		1.13 (0.95–1.36)	7 estimates
				B		1.6 (1.1–2.3)	5 estimates

^a ^ST = smokeless tobacco; ever exposure includes undefined use.

^b ^NS = never smokers; SNS = smokers and nonsmokers combined (with adjustment for smoking).

^c ^L&H = Lee and Hamling review [2]; B = Boffetta et al. review [1].

^d ^To within rounding error, as B only expressed estimates to one decimal place.

^e ^Estimated from data provided.

**Table 4:** Comparison of individual and overall (random-effects) estimates for the two reviews – pancreatic cancer

	ST use^a^						
						
Ref	Type	Exposure	Inclusion of smokers^b^	Review^c^	Sex	Relative risk (95% CI)	Comments
[26]	ST	Ever	SNS	L&H	M	1.7 (0.9–3.1)	
	ST	Ever	SNS	B	M	1.7 (0.9–3.1)	Estimates agree
							
[4]	Snuff	Ever	SNS	L&H	M	1.67 (1.12–2.50)	Estimates for NS also available
	Snuff	Ever	SNS	B	M	1.7 (1.1–2.5)	Estimates agree^d^
							
[5]	Snuff	Ever	SNS	L&H	M	0.9 (0.7–1.2)	
	Snuff	Ever	NS	B	M	2.0 (1.2–3.3)	NS not SNS
							
[27]	ST	Ever	SNS	L&H	M	0.29 (0.09–0.92)^e^	Not included by B
							
[28]	Chew	Ever	NS	L&H	M	2.82 (0.85–9.39)	Personal communication from Dr Muscat
	Chew	Ever	NS^f^	B	M	3.6 (1.0–12.8)	Estimate actually for non-current smokers
							
[29]	ST	Ever	NS^g^	L&H	M+F	1.1 (0.4–3.1)	
	ST	Ever	NS	B	M+F	1.4 (0.5–3.6)	Estimate biased as pipe and cigar smokers included in numerator only
							
[30]	ST	Ever	SNS	L&H	M+F	0.65 (0.43–0.97)^e^	Estimate for NS also available
	Chew	Ever	NS	B	M+F	0.6 (0.3–1.4)	Chew not ST; NS not SNS
							
Total				L&H		1.07 (0.71–1.60)	7 estimates
				B		1.6 (1.1–2.2)	6 estimates

^a ^ST = smokeless tobacco; ever exposure includes undefined use.

^b ^NS = never smokers; SNS = smokers and nonsmokers combined (with adjustment for smoking).

^c ^L&H = Lee and Hamling review [2]; B = Boffetta et al. review [1].

^d ^To within rounding error, as B only expressed estimates to one decimal place.

^e ^Estimated from data provided.

^f ^See comment.

^g ^Never cigarette smokers, with adjustment for other tobacco use.

**Table 5:** Comparison of individual and overall (random-effects) estimates for the two reviews – lung cancer

	ST use^a^						
						
Ref	Type	Exposure	Inclusion of smokers^b^	Review^c^	Sex	Relative risk (95% CI)	Comments
[31]	ST	Ever	NS	L&H	F	6.80 (1.60–28.5)	Not included by B
							
[3]	ST	Current	NS	L&H	M	1.08 (0.64–1.83)	
(CPS-I)	ST	Current	NS	B	M	1.1 (0.6–1.8)	Estimates agree^d^
							
[3]	ST	Ever	NS	L&H	M	1.77 (1.14–2.74)^e^	
(CPS-II)	ST	Current	NS	B	M	2.0 (1.2–3.2)	Current not ever exposure
							
[4]	Snuff	Ever	SNS	L&H	M	0.80 (0.61–1.05)	Estimate for NS also available
	Snuff	Ever	SNS	B	M	0.8 (0.6–1.1)	Estimates agree^d^
							
[5]	Snuff	Ever	SNS	L&H	M	0.7 (0.6–0.7)	
	Snuff	Ever	NS	B	M	0.8 (0.5–1.3)	NS not SNS
							
[27]	ST	Ever	SNS	L&H	M	0.69 (0.47–1.00)^e^	Not included by B
							
Total				L&H		0.99 (0.71–1.37)	6 estimates
				B		1.2 (0.7–1.9)	5 estimates^f^

^a ^ST = smokeless tobacco; ever exposure includes undefined use.

^b ^NS = never smokers; SNS = smokers and nonsmokers combined (with adjustment for smoking).

^c ^L&H = Lee and Hamling review [2]; B = Boffetta et al. review [1].

^d ^To within rounding error, as B only expressed estimates to one decimal place.

^e ^Estimated from data provided.

^f ^B only presented four estimates, but a combined result stated to be based on five. The random-effects estimate for the four estimates provided is 1.1 (0.7–1.6)

### Sources of difference between the two reviews

#### Derivation of estimates

Whereas Boffetta et al. [1] limited themselves to using RR estimates given in the source publication, we [2] calculated an estimate using available methodology [32-35] where the required RR was not provided but could be derived from data given in the publication,. We felt this necessary so as to avoid omitting relevant studies completely or, when a study provided non-independent results from subsets of the data, presenting results only for one of the subsets.

#### Restriction to smoking-adjusted estimates

As noted earlier, Boffetta et al. [1] stated that they "included only studies restricted to non-smokers and studies that included smokers but were properly adjusted for the possible confounding effect of tobacco smoking." Though, as is so often the situation in smoking and health literature, the term "non-smokers" was not defined, we have assumed that "lifelong non-smokers" (i.e. never smokers) was meant. What was meant by "properly adjusted" was also undefined, and in practice it appears that any smoking adjustment was accepted, as we could find no case of a published smoking-adjusted RR that was not included by Boffetta et al. This is not surprising since, as noted in our review [2], only a small proportion of studies took any account of daily consumption or duration of smoking. As a consequence, the smoking-adjusted data in Tables 2, 3, 4, 5 taken from our review are also not restricted on how the adjustment for smoking was carried out.

#### Studies included

We included more studies in our review [2] than did Boffetta et al [1] in theirs. Mainly these are studies [7-9,13,14,17,23,27] where the estimate required calculation, but three studies [20,21,31] appear to have been overlooked by Boffetta et al., and there is also a recent study [6] published after September 2007, the cut-off date for their literature search. There is only one study [11] included by Boffetta et al., but not by us. This study did not provide results for never smokers, and though it claimed to have presented estimates adjusted for tobacco use, this appears impossible as the authors stated that "for users of multiple tobacco products, only the primary product was recorded." A comparison of ST users with non-users of tobacco will therefore be biased by smokers being included only in the group using ST.

#### ST type

The majority of studies presented results only for one type of ST, usually either snuff specifically (typical for Scandinavian studies) or for overall ST use. A few studies provide separate RRs for snuff and chewing tobacco. The RRs we used in our meta-analyses [2] were based on overall ST use if possible, calculated if necessary from the separate results. We note that there were two studies [15,30] where results were available for snuff and chewing tobacco, and where Boffetta et al. [1] included only the results for chewing tobacco.

#### ST exposure

The great majority of the studies present only RRs for either ever use or unspecified use (which both reviews [1,2] have considered as essentially equivalent to ever use). Some studies present results for current and former use, and our procedure was to include the result for current use only if an estimate for ever use was not available or could not be calculated. Since they did not calculate RR estimates, Boffetta et al. [1] included current rather than ever use estimates for lung cancer for CPS-II [3]. However, otherwise the two sets of estimates agree as regards ST exposure.

#### Selection of results according to smoking history

There are a number of studies where RRs are available both for never smokers and for smokers and non-smokers combined, with adjustment for smoking. In the meta-analyses shown in Table 1 taken from our review [2], we have always included the smoker/non-smoker combined estimate from these studies, on the basis that they provided greater power, though our review also presents the results of meta-analyses of RRs specifically for never smokers. Boffetta et al. [1] appear not to have defined any rule here. In three such studies [5,22,30] they include results for never smokers, and in two studies [4,19] the results for smokers and non-smokers combined, without any supporting explanation.

#### Types of cancer

Where results are available by type of cancer, we have always included estimates for the total cancer being considered, but this is not the case for Boffetta et al [1]. Thus whereas, for Table 2, we [2] include RRs for overall oropharyngeal cancer, if available, only considering cancers of particular regions of the oropharynx if these were the only data presented, Boffetta et al. omitted relevant results for pharynx cancer in one study [18] and presented RRs separately for mouth and tongue cancer in another [11], a study in which results were also available for a number of other regions of the oropharynx. For oesophageal cancer, there are two studies [22,25] where Boffetta et al. included results only for squamous cell carcinoma, omitting those for adenocarcinoma, despite the other studies in their analysis only presenting results for overall oesophageal cancer.

#### Unnecessary inclusion of a confounded result

In one study [29] Boffetta et al. [1] selected a RR for never cigarette smokers in which ST users who may also have smoked pipes or cigars were compared with user of no tobacco at all. We [2] preferred an estimate using those who had only used ST as the numerator, to avoid bias from pipe and cigar smoking.

#### Meta-analysis

Although Boffetta et al. [1] did not define their method, it appears that they used random-effects estimates as we [2] did, since for three of the cancers we calculated the overall estimate from their data under this assumption and obtained the same answer they did. We could not check this for lung cancer as they presented only four individual study RRs, but gave an overall estimate based on five.

### Effect of the differences

The fact that for some studies we calculated RRs from data available, combining evidence from data subsets to make fuller use of the data, and that we applied a consistent rule for choosing between RRs for never smokers and RRs for smokers and non-smokers combined has led to differences between the RRs we include in our meta-analyses [2] and those used by Boffetta et al. [1]. Some of the differences are minor, but it is apparent that where there are substantial differences, they are always in one direction, with our systematic and arguably more complete analysis providing lower smoking-adjusted RRs than theirs.

Five particular cases deserve comment. For oesophageal cancer, Zendehdel et al. [22] reported never smoking RRs of 3.5 (95% CI 1.6–7.6) for squamous cell carcinoma, 0.2 (0.0–1.9) for adenocarcinoma, as well as smoking-adjusted RRs of 1.0 (0.8–1.4) for squamous cell carcinoma and 1.0 (0.6–1.5) for adenocarcinoma. Our smoking-adjusted RR shown in Table 3 was derived by combining the last two relative risks to give 1.00 (0.79–1.27), whereas Boffetta et al. [1] used only the first, high, estimate of 3.5 (1.6–7.6).

For pancreatic cancer, the RR selected by Boffetta et al. [1] for the study by Luo et al. [5] was that for never smokers (2.0, 1.2–3.3) and not the smoking-adjusted estimate (0.9, 0.7–1.2) we [2] used. In contrast in the Norway cohorts study [4] both reviews used the smoking-adjusted RR of 1.67 (1.12–1.50), Boffetta et al. here not selecting the lower never smoker estimate of 0.85 (0.24–3.07). Also, as noted above for the study by Alguacil and Silverman [29], Boffetta et al. selected a higher, but biased, RR of 1.4 (0.5–3.6) when we used an estimate of 1.1 (0.4–3.1).

For oropharyngeal cancer, in the study of Kabat et al. [16], Boffetta et al. chose a never smoker estimate of 2.3 (0.70–7.3) rather than the smoking-adjusted RR of 1.1 (0.81–1.53) we [2] used, while in the study of Lewin et al. [18] they chose an estimate of 1.4 (0.8–2.4) for oral cancer overlooking one of 0.7 (0.4–1.3) for pharyngeal cancer, rather than the combined oropharyngeal RR we used of 0.98 (0.63–1.50).

Exclusion of relevant studies, and in one case [11] inclusion of RRs that seem not to be smoking-adjusted, have also contributed to the difference. This is considered further below, where we comment on the four cancers in turn.

#### Oropharyngeal cancer (Table 2)

Based on the 13 individual estimates provided by Boffetta et al. [1], we calculate the random-effects meta-analysis estimate as 1.82 (1.14–2.90), agreeing, to one decimal place, with their figure of 1.8 (1.1–2.9). This is markedly higher than the combined estimate of 1.36 (1.04–1.77) based on the RRs we included. Eliminating the RRs from the Stockwell and Lyman study [11], which appear not to be smoking adjusted, would reduce Boffetta et al.'s estimate to 1.54 (0.99–2.38) and further, for reasons noted earlier, replacing their RR estimates from the studies by Kabat et al. [16] and Lewin et al. [18] by ours would reduce the combined estimate further, to 1.36 (0.94–1.98), making it similar to ours [2]. Adding in the extra studies we included [6-9,13,14,17,20,21] has little effect on the overall estimate.

**Figure 1:**
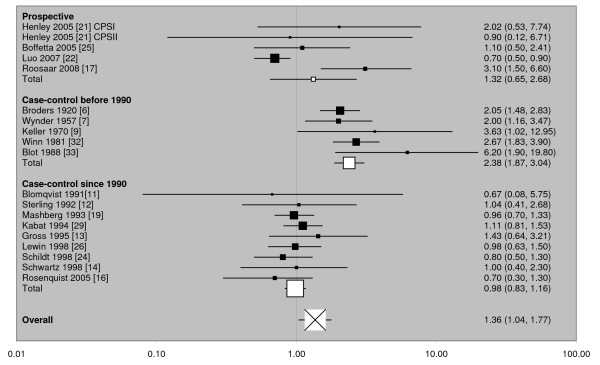
**Variation in RR of ST-associated oropharyngeal cancer by study type and period of publication**. For each of 19 studies, separated by study type and, for case-control studies, by period of publication, the individual study RR and 95% CI estimates, taken from the Lee and Hamling review [2] (see also Table 2), are shown numerically and also graphically on a logarithmic scale. In the graphical representation, the RR is indicated by a solid square, with the area of the square proportional to the weight (inverse-variance) of the estimate. Also shown are the combined estimates, derived by random-effects meta-analysis, for the three subgroups and overall. Here the sizes of the four squares corresponding to the RRs are also proportional to the weight of the estimate, though the constant of proportionality differs from that for the individual RRs.

The conclusions of Boffetta et al. [1] regarding the role of ST, as used in Western countries, on the risk of oropharyngeal cancer fail to take account of the additional evidence in our review [2] and elsewhere [36] that any excess risk essentially vanishes when attention is limited to studies that have adjusted for alcohol as well as smoking. While there may have been some effect in the past of ST as used in the USA, Boffetta et al. refrain from commenting on the fact that RR estimates have declined markedly over time. This decline is illustrated clearly in Figure 1 where the consistent (heterogeneity p = 0.34) evidence of an increase seen in case-control studies published before 1990 contrasts sharply with the consistent (heterogeneity p = 0.93) total lack of evidence of an increase in case-control studies published more recently. The prospective studies, each of which involves a long-term follow-up period starting many years ago (1959–1972, 1982–2000, 1966–2001, 1978–2004, 1973–2002 for the five studies in Figure 1 in order), give results that are heterogeneous (p = 0.004) and suggest an intermediate increase. (It should be noted that for other cancers the data are too limited to allow useful comparison between studies published before and after 1990)

#### Oesophageal cancer (Table 3)

Here the meta-analysis estimate we calculate based on the five RRs given by Boffetta et al. [1] is 1.57 (1.09–2.28), matching the estimate they give, of 1.6 (1.1–2.3). The difference between this estimate and ours (1.13, 0.95–1.36) is virtually wholly due to the RRs selected for the study by Zendehdel et al. [22], as replacing their estimate by ours for this study reduces their combined estimate to 1.10 (0.91–1.34), similar to our estimate of 1.13 (0.95–1.36).

We consider that there is no convincing evidence that ST increases the risk of oesophageal cancer. The results from the Zendehdel et al. study [22] suggesting an increase specifically for never smokers as regards squamous cell carcinoma clearly need confirmation by other studies before any reliable conclusion can be drawn.

#### Pancreatic cancer (Table 4)

Based on the data of Boffetta et al. [1], our combined estimate is 1.57 (1.09–2.25), agreeing with their 1.6 (1.1–2.2), but markedly higher than the estimate based on our data, of 1.07 (0.71–1.60). Amending, for reasons discussed above, the estimates for the studies by Luo et al. [5] and by Alguacil and Silverman [29] to the ones we used would reduce their overall figure to a non-significant 1.25 (0.83–1.88).

Our estimate of 1.07 (0.71–1.60) is somewhat lower than this due to inclusion of the low estimate we calculated from the Williams and Horm study [27]. As discussed elsewhere [37], where there is a fuller discussion of the evidence on this cancer, some objections can be made about this study. However the conclusion from our review [2], that any effect of ST on pancreatic cancer has not been clearly demonstrated, seems justified by the data whether or not results from this study are included.

#### Lung cancer (Table 5)

We cannot evaluate the lung cancer meta-analyses of Boffetta et al. [1] due to their only providing four of the five individual RRs they used. However, their RRs are quite similar to ours for the four studies where comparison is possible. Our analysis [2] also includes a high RR from one study [31] and a low RR from another [27] and we agree that an association has not been demonstrated.

## Discussion and conclusion

We believe that our review [2] offers a more robust meta-analysis of the data than previously conducted by Boffetta et al. [1] for a number of reasons. One reason is the use of derived as well as published estimates, which adds considerably to the data available for analysis, an approach which might be improved still further by obtaining results for those studies which merely reported their findings non-quantitatively, e.g. as "no significant association." Other reasons include ensuring that all the RRs used were in fact adjusted for smoking, and the use of a pre-defined systematic procedure to decide which estimates to include in the meta-analysis. The differences in procedures had the largest effect for pancreatic cancer and oesophageal cancer. For pancreatic cancer, significant increases for never smokers in one study [5] and for smokers and non-smokers combined in another study [4] were selected by Boffetta et al., ignoring estimates showing a lack of any increase at all for smokers and non-smokers combined in the first study [5] and for never smokers in the second [4]. For oesophageal cancer, given results for one study [22] which showed a significantly increased RR among never smokers for squamous cell carcinoma but no increase at all among never smokers for adenocarcinoma, or among smokers and non-smokers combined for either cell type, Boffetta et al. elected to include only the significant RR, despite the inherent bias from such a procedure. For oropharyngeal cancer, although Boffetta et al. recognized the lack of evidence of a relationship for studies conducted in Scandinavia, their claim of an effect for ST as used in the USA fails to recognize that this is no longer seen in more recent studies.

As a result of using a more systematic and more inclusive process we believe that our analysis [2] provides a more accurate estimate of any relationship of ST with risk of cancer. Previous claims of significant increases for oropharyngeal, oesophageal and pancreatic cancer with risk increases of 60% to 80% for each cancer [1], appear unjustified when more appropriate meta-analyses are conducted. For the oesophagus and pancreas the estimated risk increases based on smoking-adjusted data should be more like 10% and not statistically significant, while for oropharyngeal cancer we estimate the increase to be a marginally significant 36% when all the data are considered, and to be zero when attention is restricted to studies published since 1990.

Boffetta et al. [1] also claimed that the cancer risk of ST users is only "probably" lower than that of smokers. Given that, for the four cancers they consider, the RRs they estimate are substantially lower than seen for smoking (particularly for lung cancer where their estimate of a 20% increase for ST compares with an estimated increase of about 2000% for current smokers and 1000% for former smokers), it is unclear why they did not accept that the risk for ST users is definitely much lower than for cigarette smokers. This conclusion is even more evident using our more appropriate risk estimates for ST, and in our review [2] we estimate that attributable deaths from smoking-related cancers would be almost 100 times lower, if smokers instead had the risk of ST users.

While, as discussed in our review [2], the evidence we have considered has many weaknesses and, as Boffetta et al. state in their review [1], the health effects of ST products need to be better characterized, we feel it is important that appropriate inferences are drawn from the data that are available so as to put the likely cancer risks from use of ST into a proper perspective versus the risks of smoking cigarettes. This is important given that some public health authorities see a potential role for ST in tobacco harm reduction [38-40]. We also feel that this investigation underlines the advantage of a pre-defined systematic procedure for conducting meta-analyses. It is essential that all relevant data should be used, and that clear rules should be present for choosing between alternative estimates from the same study.

## Abbreviations

CI: 95% confidence interval; CPS-I: American Cancer Society Cancer Prevention Study I; CPS-II: American Cancer Society Cancer Prevention Study II; RR: relative risk; ST: smokeless tobacco.

## Competing interests

PNL, founder of P.N. Lee Statistics and Computing Ltd., is an independent consultant in statistics and an advisor in the fields of epidemiology and toxicology to a number of tobacco, pharmaceutical and chemical companies. JH works for P.N. Lee Statistics and Computing Ltd.

## Authors' contributions

PNL conceived and planned the study and carried out the literature search. PNL and JH jointly extracted the estimates and conducted the meta-analyses. The text and tables were drafted by PNL and checked by JH. Both authors read and approved the final manuscript.

## Pre-publication history

The pre-publication history for this paper can be accessed here:

http://www.biomedcentral.com/1471-2407/9/256/prepub
